# Teach the Unteachable with a Virtual Reality (VR) Brain Death Scenario – 800 Students and 3 Years of Experience

**DOI:** 10.5334/pme.1427

**Published:** 2025-01-28

**Authors:** Anna Junga, Pascal Kockwelp, Dimitar Valkov, Henriette Schulze, Philipp Bozdere, Ole Hätscher, Helmut Ahrens, Bernhard Marschall, Benjamin Risse, Markus Holling

**Affiliations:** 1 Institute of Medical Education and Student Affairs, University of Münster, Münster, Germany; 2 Institute for Geoinformatics and Institute for Computer Science, University of Münster, Münster, Germany; 3 Faculty of Natural Sciences and Technology Saarland University, Germany; 4 Institute for Society and Digital Media, Münster University of Applied Sciences, Münster, Germany; 5 Department of Psychology, University of Münster, Münster, Germany; 6 Institute of Education and Student Affairs, University of Münster, Münster, Germany; 7 Institute for Geoinformatics and the Institute for Computer Science, University of Münster, Münster, Germany; 8 Department of Neurosurgery, University Hospital Münster, Münster, Germany

## Abstract

**Introduction:**

Traditionally, clinical education has combined classroom theory with hospital-based practical experiences. Over the past 50 years, simulation-based training, particularly virtual reality (VR), has gained prominence for its flexibility and scalability. This article describes the development, implementation and evaluation of a VR-based brain death diagnostic training module at the University of Münster over a three-year period.

**Methods:**

A multidisciplinary team developed the VR scenario to simulate a realistic intensive care unit, in line with German guidelines for brain death diagnosis. The module includes a tutorial and a preparatory video podcast to accommodate varying levels of VR experience. The course maintained its former small-group format, integrating VR to replace a manikin-based brain death examination. A randomized pilot study compared the traditional and VR-based approaches.

**Results:**

Feedback from over 800 students indicated a strong preference for VR training, with a significant increase in perceived competence in brain death diagnosis. The VR module also increased the individual training time and provided more varied clinical scenarios than traditional methods. Continuous feedback led to iterative improvements, including reflex simulations and improved hardware management.

**Discussion:**

The VR-based training was well received, demonstrating its potential to revolutionize medical education by providing immersive, realistic simulations. Challenges such as initial hardware adaptation and high personnel costs were addressed through comprehensive tutorials and structural adjustments. The success of this module has led to the development of additional VR courses, optimizing the use of hardware and justifying the initial investment.

**Conclusion:**

The integration of VR into medical education at the University of Münster has proven effective, enhancing student engagement and competence in brain death diagnosis. The positive outcomes suggest a promising future for VR in medical education, highlighting the importance of innovative tools in the preparation of future medical professionals. Efforts are continuing to broaden the application and accessibility of VR-based training.

## Introduction

Traditionally, clinical education was provided in classrooms to teach theory and in hospitals to gain practical experience [1]. With the growing significance of practical skills and competencies, simulation has become increasingly important over the last 50 years [2]. Simulation-based training can be categorised based on the setting used to recreate a certain aspect of medical practice, namely manikin-based, actor-based, role-playing-based, and computer-based-training [3]. All these settings have their advantages and disadvantages. While all these settings can provide a safe learning environment, the aspect of repeatability and standardisation is of particular interest in VR; even special (complex or otherwise challenging) scenarios can be presented. The cost effectiveness may also be more favourable than other simulation training in the long run [3]. However, computer-based training has become particularly important due to the rapid development of immersive technologies and especially virtual reality (VR) hardware. It can potentially transform educational experiences while being more flexible and scalable than any other simulation-based training [3,4].

Attempts to use VR for medical education started two decades ago [5]. Since then, several VR tools have been developed for training purposes, mainly in the areas of surgical simulation, 3D anatomy training, emergency training and communication training, as described in detail below. By far the most prominent class of medical VR training is surgical simulation, which aims to improve practical skills [6] and often incorporates haptic hardware to provide realistic user feedback [7]. Other examples of surgical training using VR [8,9,10,11] and dedicated reviews for neurosurgery and laparoscopic surgery are published in two series [12,13] respectively. In a different approach, 3D models are combined with VR to train anatomical skills and spatial reasoning [14]. These models are often generated from real radiological images to provide a realistic clinical context [15]. VR is also particularly suitable for training emergency situations involving acute events or mass casualties [16,17]. These simulations often allow for multi-user experiences to enhance immersion. VR can also be used to train soft skills such as communication strategies or to train empathy [18,19]. A variety of professional simulations have been introduced in the past, such as nursing training [20] or dentist simulations [21]. A comprehensive review of the use of VR simulations in medical education can also be found in [22].

Several learning theories have been applied to VR education, but more research is needed to justify their use and develop effective methods [23]. Experiential learning theory is particularly relevant, emphasising that learning is most effective when learners engage in all stages of experience, reflection, abstract thinking and experimentation [24]. Constructivism, which suggests that learners construct knowledge through active engagement and reflection, is also often referred to [25]. VR supports both theories by providing immersive, interactive environments for exploration and skill application [23]. In addition, gamification elements in VR can increase motivation, engagement and satisfaction [26] and as a potentially affective learning method, VR can provide emotionally triggered learning and recognition [27,28].

Most of the training simulations mentioned above focus on practical clinical skills (e.g. surgery) or teaching complex medical knowledge (e.g. anatomy). Diagnostic skills and clinical reasoning are under-represented because they are still mainly taught using traditional techniques such as manikins, actors or role-playing. However, practical and ethical limitations prevent training on a variety of clinical cases using these approaches. The examination of brain death is a prime example, as neither actors nor manikins offer sufficient flexibility to allow unbiased training (i.e. actors cannot simulate brain death symptoms and manikins cannot simulate non-brain death outcomes). In addition, certain relevant diagnostic steps cannot be performed in either setting.

A manikin-based brain death examination as part of a model project on organ transplantation has been standard for teaching at the medical faculty of the University of Münster for many years. Although the topic is not part of the national curriculum, students have a whole week to deal with the complex topic of organ donation and transplantation in various course formats. In addition to specialized lectures and dialogue with transplant patients, all students undergo a full day of skills training [29,30]. Moreover, since organ donation is an important but complex topic and is legally based on the consent solution in Germany, training future professionals in this area is of utmost importance. Consequently, the organ transplantation course aims to provide a safe and comfortable environment for individuals to ask questions and learn how to advise relatives in the case of potential organ donation.

Within this skills training programme all communications-and-interaction-trainings were demonstrated using simulated patients. The practical implementation of the brain death examination was based on a small group demonstration on one manikin. However, it was not economically viable to provide enough manikins for each student, especially as the reflexes of a (non-mechanical) manikin would always be negative. Positive reflex responses and behaviour could only be discussed theoretically.

The aim was to develop a more immersive and sustainable way to teach this highly relevant topic, reduce student anxiety, and provide information about the process of brain death diagnosis. An additional challenge was to fully integrate this tool into the compulsory curriculum to maximize the number of students accessing this innovative teaching method.

In a first attempt, we questioned whether VR could be a viable tool for teaching about brain death. In a second attempt, we aimed to identify and address shortcomings, develop improvements and evaluate these updates in an iterative process over the last three years.

## Methods

### Development

In an inter-professional dialogue, a VR-based software solution was identified as a promising innovative solution. A medical didactics team and medical experts were responsible for developing a suitable scenario and integrating the new VR training into the current curriculum. The virtual brain death diagnosis scenario was developed in collaboration with experts from the computer science department and technical experts developed the training software according to medical specifications and revised the software several times.

A standard intensive care unit room was chosen as the VR setting, equipped with all the tools required to perform a brain death examination according to the current German guidelines [31]. In brief, the patient lies in a hospital bed in the centre of the room and is connected to a ventilator.

Consultation with medical specialists and usability testing with VR-inexperienced subjects took place at regular intervals. As part of this, improvements were made to the level of detail within the virtual environment to improve medical congruence (e.g. bruises and bandages as catheters and wires were added, a second clock was implemented within the field of view of the patient [32]). Additional adjustments affected the interactions; gestures were aligned with standard medical practices, and trigger points (these are used to determine the animation of various movements based on localisation, despite the limited freedom of movement of the hands on the real controllers e.g. the grasping gesture at the eye is implemented as a tweezer grip, were repositioned. Further technical details are described in Kockwelp et al. [33].

In the fourth year of medical school, students undertake a project week on organ donation and transplantation. This begins with several relevant lectures (one specific to brain death examination). In addition to discussion rounds with transplanted patients and living donors, the ‘Tx module’ simulation day takes place at the training hospital. The structure of the existing ‘Tx Moduel’ course was retained: Students attend the course in small groups of six. After an introductory seminar, one representative from each group conducts a dialogue with a simulation patient who is to be informed that he or she is suffering from organ failure and, therefore, needs a donor organ. The remaining members of the small group observe this conversation from an adjoining room and subsequently provide feedback. This is followed by the part of the course on diagnosing brain death that was taught previously with the whole group of six on a manikin (see Figure 2 A+B). Then there are two further dialogues performed by one student per group of six as described: Giving the bad news of a patient’s brain death to the family and discussing whether the patient has previously expressed or documented a preference for organ donation. The learning objective for this whole week is to expose students to the relevant but emotionally complex topic of organ donation and transplantation, and to reduce distress and anxiety. For the brain death module in particular, the course should help to demonstrate and reinforce the procedure to be followed in the event of possible brain death, so that students will remember at least the general protocol years later.

The new VR-based module replaced the section of the manikin-based brain death examination. In addition, a tutorial was added to the course (see the next section *Implementation of the pilot*). To avoid interrupting the narrative flow mentioned above, the tutorial does not take place immediately before the VR simulation, but before the first interaction with the simulated patients. Due to the different levels of experience with VR (from very experienced to no experience), the hardware is explained at the beginning of the VR part.

**Figure 1:**
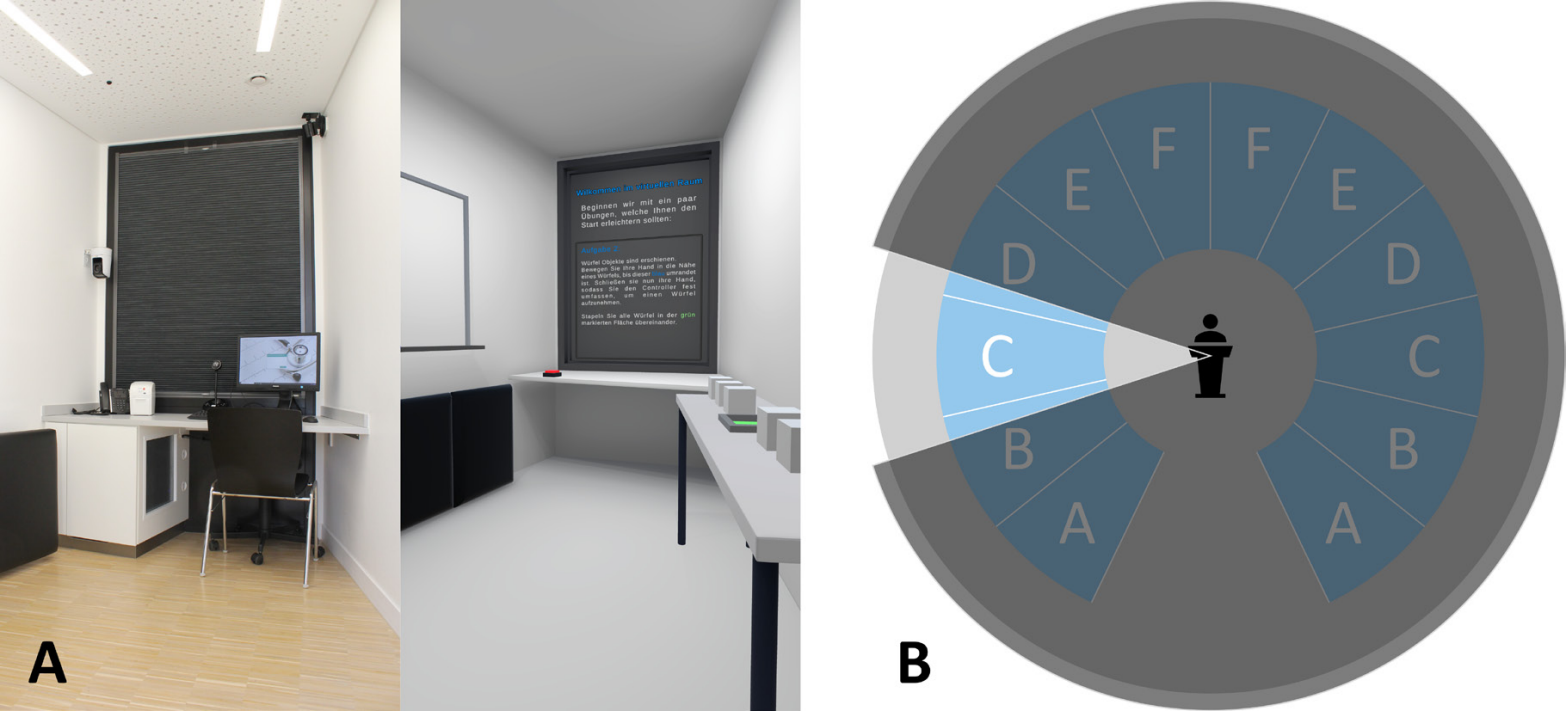
Illustrative comparison of the real course room (**A**, left) and the virtual tutorial course room (**A**, right) as well as schematic representation of the room layout and the possibility of supervision **(B)**.

A suitable location for the VR course was the existing training facility “LIMETTE” at the University of Münster, which consists of 12 identical rooms that can be viewed from a central control room through a one-way mirror. The rooms are connected by an intercom system, which allows the students in each room to speak to each other (Figure 1).

### Implementation of the Pilot

For the pilot, six rooms were equipped with Steam’s Valve Index head-mounted displays (HMDs) and high-performance computers to run the VR software developed. These rooms had to be structurally adapted, e.g. additional sockets had to be installed, and the lighthouses (three-dimensional navigation devices) were permanently installed. A mechanism was added to the computer cabinet doors to keep them slightly open – full opening was not practical due to the risk of injury. To optimise supervision and staffing, additional screens were installed in the control room to show the students’ VR perspective alongside the real rooms.

Based on the feedback from the user tests, a VR tutorial was added to the application (see Figure 1, A right). This allowed users to practice all necessary interactions in a modified form, such as opening the pen instead of turning on the flashlight, without prior knowledge of the application’s content. A standard VR teleportation metaphor (designed for the Valve Index Knuckles controllers) was also integrated to allow unrestricted and efficient movement, so that all students were able to navigate in virtual spaces that were potentially larger than the real space.

**Figure 2:**
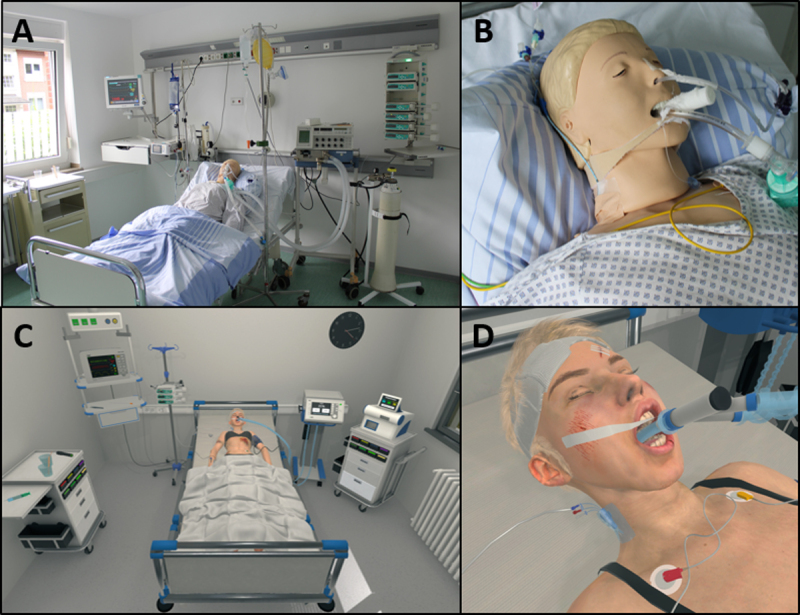
A Moc ICU used in the old course design with overview of the whole room and **B** close-up of the patient; **C+D** identical view in the virtual setting.

During the course, students are given the following task: A 28-year-old female patient has suffered significant head trauma in a quad bike accident. Brain death is suspected and needs to be investigated (task sheet in supplementary material 2). For this, the students are in the role of a physician in an intensive care unit. When they put on the VR headset, a young, intubated patient is lying in a bed in the centre of the room (Figure 2 C+D; Supplementary material 2, Figure 3). The hospital room contains all the necessary equipment to carry out a brain death examination according to the current German guidelines [31].

**Figure 3:**
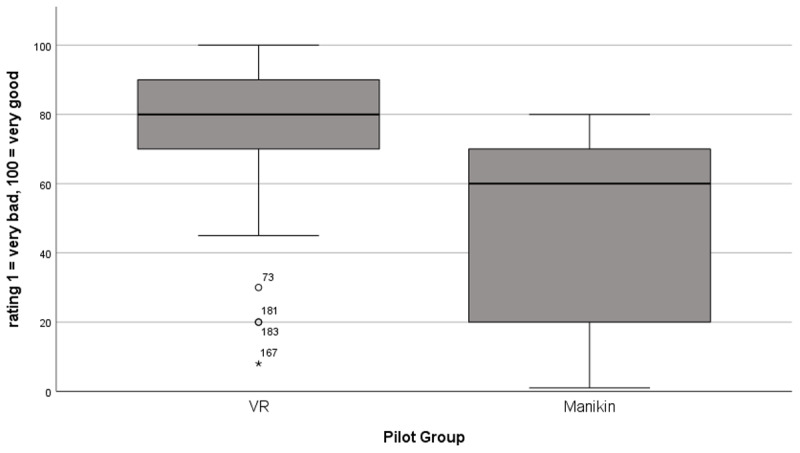
Boxplot of the global rating analysed according to the VR and manikin study groups, n = 84 (n_VR_ = 42, n_MK_ = 42).

In addition to considerations of teaching unit capacity and coordination with the other departments involved in the module, several hardware and software tests were conducted in advance of the course.

57 of the students piloted this concept in VR in July and October 2021, the other 62 continued to use the old teaching structure with the manikin. The students were distributed alternately in groups of 6 (see below for further details). Randomised distributions were used to allow unbiased statistical evaluations. The students were given a lecture on brain-dead diagnostics as a video podcast on the same topic. In addition, all students in the VR group completed a tutorial in VR to familiarise themselves with navigation and interaction immediately prior to the course. Tutors provided technical support during the tutorial and the course if needed. The course was then run twice a year (see Table 1).

**Table 1:** Demographic overview over the study groups.


STUDY GROUP	TIME	PARTICIPANTS QUESTIONNAIRE-PRE/POST-QUESTIONNAIRE	RESPONSE RATE IN %	AGE M(±SD)	GENDER (F/M/D*)

1 (pilot)	July 2021	**119** – 101/86	84,9	23,9(±3,5)	74/27/0

1.1 (pilot)	October 2021	**123** – 114/84	92,7	24,3(±3,4)	76/38/0

2	January 2022	**137** – 124/106	90,5	23,4(±3,0)**	71/51/1

3	July 2022	**129** – 111/57	86,0	23,9(±3,1)**	71/38/1

4	January 2023	**110** – 110/89	100	23,5(±3,4)	65/45/0

5	June 2023	**122** – 121/112	99,2	24,5(±3,3)	82/38/0

6	January 2024	**118** – 117/117	99,2	23,8(±3,1)	74/42/1

overall	July 2021–January 2024	**858** – 798/651	93,0	23,9(???)	513/279/3


*Option available from Jan 2022. **approximate, as option >30 years was available.

#### Survey and statistics

In each semester, a pre- and post-survey was conducted immediately during the course. The survey was administered online using LimeSurvey (version 6.6.4) on the institution’s computers. The questionnaire was adapted due to software updates (e.g. added questions for tutorial and video podcast), the latest version can be found in [supplement 1]. In the first version for the pilot group, students were asked about demographics as well as an overall rating of the course from 1–100 and which course format they would prefer as a choice between ‘VR’, ‘manikin’ and ‘no preference’. The extended version of the questionnaire includes, in addition to a global rating (1–100) and content-specific questions about the podcast and tutorial (5-point Likert scale), questions about the importance of the learning unit and the students’ subjective assessment of the learning process (5-point Likert scale) [all questions in detail are available in supplement 1]. As part of the follow-up study, students’ examination results, cybersickness and BIG-5 (psychological questionnaire for an additional research project, [34]) questionnaire data were also collected, but are not included in the present study.

The software used for statistical analyses was Microsoft Excel version 2409, build 18025.20140) and IBM SPSS version 29.0.0.0 (241). The Microsoft Excel function (= ROUND(RAND()*(1–0) + 1;0)) was used to divide the affected students into 2 groups (VR or manikin). An independent samples t-test was performed to identify significant differences in the ratings between the two study groups (pilot). A χ^2^ test with Bonferroni correction (α = .0167) was performed (pilot) to identify differences in stated preference for a method (VR or manikin). Otherwise, α < 0.05 was considered significant. A paired samples t-test was performed to identify significant differences in subjective competence gain over time in the VR group (cohort 6), an independent t-test to measure differences in examination time required with and without the additional podcast (pilot vs. cohort 6).

The protocol was approved by the local ethics committee of the University of Münster, Germany (Ethik-Kommission Westfalen-Lippe: # 2022–736-b-S; 09.11.2022). Informed consent was obtained from all participants.

## Results

### Establishing the VR course

In total, almost 800 students have participated in the hybrid (n = 242) or fully VR-based (n = 616) course on brain death diagnosis since July 2021 (Table 1). A number that would not be feasible using real patients due to ethical incompatibilities and due to the rarity of the condition.

The response rate for the specific evaluation across all semesters was 93,0%.

The students’ previous experience with VR was surveyed at several points in time. Overall, only 0.5% to 1.8% (depending on the cohort) of students reported having regularly used a VR headset before the course. Among students taking a VR course for the first time, 69.4% to 77.3% stated that they had never used a VR headset. The trend is slightly declining in our evaluation over time, which is in line with the rising commercial availability of VR headsets.

**Figure 4:**
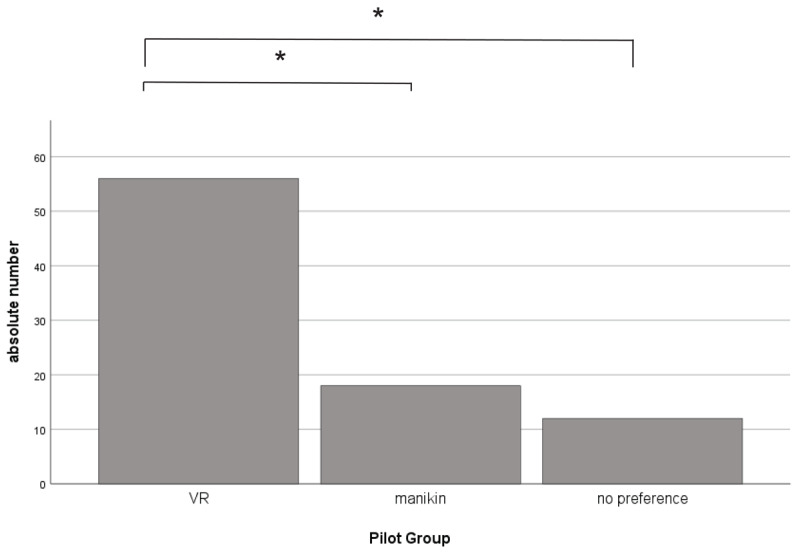
Preference for teaching method, VR n = 56; manikin n = 18; no preference n = 12; overall n = 86, significant differences as stated in the text.

The pilot students (see Table 1) were asked to rate their course format. The result shows a significantly better rating of 74.4 (±22.2) [max. 100] points for the VR course and 47.4 (±28.9) [max. 100] points for the manikin-based course (t(82) = 4.79, p < .001), n = 84) (Figure 3). All students were asked which course format they would prefer in the future (Figure 4). The students clearly favoured ‘VR’ with 65.1% of the vote, ahead of ‘manikin’ with 20.9%. 14.0% of students stated that they had no preference. Significant differences were found between ‘VR’ and ‘manikin’ (χ^2^(1, N = 86) = 19.51, p < .001) as well as ‘VR’ and ‘no preference’ (χ^2^(1, N = 86) = 28.47, p < .001). No significant differences were found between ‘manikin’ and ‘no preference’ (χ^2^(1, N = 86) = 1.2, p = .273).

Based on this result, the course was firmly integrated into the curriculum – six additional VR headsets were invested in so that the course could be offered to the entire semester cohort (12 working units for parallel training).

### Improving the VR scenario

Based on the evaluations and the informal feedback that some students had minor but relevant difficulties, particularly with the hardware, two further changes were introduced for subsequent semesters.

In the pilot, it was only possible to request help from the course instructor by gestures, as the auditory connection to the control room can only be established by the course instructor. Therefore, in 2022, a button was implemented to allow the student to send an optical signal via VR software to request help. Secondly, a central monitoring system for remote control of all software applications was installed to complement the existing intercom system based on the software *Veyon* (Veyon Solutions, Chemnitz, Germany, version 4.8.2). The support staff was also optimised from five to three people – one for the control system and timekeeping, one for local support, and one for introduction and flexible tasks. These activities can also be carried out by trained student tutors, providing significant economic benefits compared to academic staff.

Furthermore, a second video podcast (Supplementary Material 4 in German language) was developed to explain the hardware (e.g. how to adjust the headsets or controllers individually) to the students before class. Watching the podcast became a prerequisite for the course. This was assessed as good comprehension (5,17 ± 0.76 [out of 6]). The overall rating of the video podcast was 79,18%. The students also evaluated the tutorial. In the most recent run (latest version: winter term 2023/2024), students rated it as good to very good with a score of 82,4% ± 16,87 (mean ± SD; n = 117). A comparison of the pilot (January 2021; available: VR Tutorial, no hardware podcast) with the last run of the course (January 2024; available: VR tutorial + hardware and medical podcast) showed a considerable reduction in the time required for the simulation from an average of Mean_2021_ = 17:03 min in 2021 to Mean_2024_ = 12:53 min in 2024.

To make the course even more realistic, further design adjustments were made to the software (e.g., head bandage, bruises, urinary catheter, central venous catheter, etc.; see Figure 2C+D). At this point, the virtual patient was also able to show reflexes for the first time during the examination, indicating residual brain activity. The presence or absence of reflexes was randomised to prevent students from drawing conclusions about the expected outcome of the examination based on the experiences of other students. To train ‘clinical reasoning’, all students had to make a diagnosis at the end of the simulation. The decision-making was implemented by using virtual buttons next to the exit of the room.

In the most recent course, the students were asked to assess their subjective competence in brain death diagnosis. At the time (t0), all students had watched the preparatory podcast (medical and hardware), and some (65,3%) had also attended the lecture. The mean score for subjective competence was (M ± SD) 2.21 (±0.85) [scale 1–5]. After the brain death simulation had been carried out (t1), the students were surveyed again. The score was significantly better at 2.98 (±0.97; n = 91), t(90) = –6,69, p < 0,001.

Further modifications were made to the structural requirements. As a result of repeated hardware failures and various inspections, the VR headset connections and the ventilation of the high-performance computers have been adjusted. All changes can be seen in Suppl. Material 3.

## Discussion

The integration of the VR Brain Death course as described above demonstrates the enthusiastic reception of the concept at the University of Münster and marks a promising path for future VR software development and integration. In addition to the improved rating, a lower variance in ratings was also achieved, suggesting that the course is of consistently better quality than the manikin variant and may be less dependent on external factors. This may also prove to be a significant advantage in the future development of virtual reality-based assessment scenarios, as suggested by Pottle and Mistry [3,35]. While adjustments were necessary to enhance the course’s efficiency and ensure sustainable use of hardware, the positive response confirms its viability. Although students initially faced challenges in adapting to the new hardware and learning environment, the growing popularity of VR hardware in general, as well as innovative learning media such as the video podcasts and tutorials provided, proved instrumental in overcoming these hurdles. Similar to the well-established Flipped Classroom concept [36], video tutorials or hands-on VR-tutorials that are presented prior to the VR course can enhance the learning process, reduce cognitive load, and improve the user experience [37,38].

The potential added value of tutorials prior to VR interventions remains largely unexplored; this is an avenue that merits further investigation.

Notably, the implemented adjustments have substantially reduced throughput time, and ongoing evaluations aim to identify further opportunities for improvement. By making structural changes and addressing inadequate computer ventilation, potential hardware failures and associated costs have been pre-emptively mitigated. Although student preparation has improved substantially over time, there is still a desire for direct student feedback. This is being followed up and a debriefing session will soon be introduced into the course. Automatic feedback by the software would also be an interesting option to give the students the chance to reflect directly. VR with direct feedback or debriefing might be beneficial in simulation-based education [39].

A limitation of this long-term observation is that conditions have changed slightly over time due to adjustments. In addition to internal adjustments to the software and learning environment, there has also been a notable increase in familiarity with VR within the commercial game studio industry [40]. The large differences in the ratings of the manikin-based scenario may also indicate that possible improvements to this course variant could have improved the overall rating. Not all questions, such as the subjective assessment of long-term recall, were asked from the outset, so a comparison with the manikin group is not possible. Nevertheless, we are confident that this study has contributed to the literature on VR in medical education.

Looking ahead, the central challenge is to ensure effective dissemination of the software. While certain elements are tailored to local facilities and existing hardware, initiatives such as a collaborative exchange could provide a solution. This would involve our group making the software freely available, allowing other groups to adapt it for diverse hardware systems at their universities, and potentially leading to open access availability. However, international use may require language and guideline adaptations.

Despite the initial high personnel and acquisition costs associated with VR implementation, its integration into teaching has proved invaluable. The ability to train individual skills and clinical reasoning individually, which are often overlooked, underlines its significance. The success of this project has paved the way for several other VR courses, with the site currently hosting five different programmes. This diversified use ensures optimal hardware utilisation, ultimately rationalising the acquisition costs.

## Data Accessibility Statement

The datasets used and analysed during the current study are available from the corresponding author on reasonable request.

## Additional Files

The additional files for this article can be found as follows:

10.5334/pme.1427.s1 Supplementary Material 1. Questionnaires used.

10.5334/pme.1427.s2 Supplementary Material 2. Task sheet VR course.

10.5334/pme.1427.s3 Supplementary Material 3. Table of adjustments during time.

10.5334/pme.1427.s4 Supplementary Material 4. Hardware tutorial podcast for “Valve Index” by Steam, revised and updated in the BMBF research project ‘meditrain’, German audio only.

## References

[1] van Way CW. Thoughts on Medical Education. Mo Med. 2017; 114(6): 417–8. 30228651 PMC6139963

[2] McGaghie WC, Issenberg SB, Petrusa ER, Scalese RJ. A critical review of simulation-based medical education research: 2003–2009. Med Educ. 2010; 44(1): 50–63. DOI: 10.1111/j.1365-2923.2009.03547.x 20078756

[3] Pottle J. Virtual reality and the transformation of medical education. Future Healthc J. 2019; 6(3): 181–5. DOI: 10.7861/fhj.2019-0036 PMC6798020 31660522

[4] Baniasadi T, Ayyoubzadeh SM, Mohammadzadeh N. Challenges and Practical Considerations in Applying Virtual Reality in Medical Education and Treatment. Oman Med J. 2020; 35(3): e125. DOI: 10.5001/omj.2020.43 32489677 PMC7232669

[5] Mantovani F, Castelnuovo G, Gaggioli A, Riva G. Virtual reality training for health-care professionals. Cyberpsychol Behav. 2003; 6(4): 389–95. DOI: 10.1089/109493103322278772 14511451

[6] Blackburn SC, Griffin SJ. Role of simulation in training the next generation of endoscopists. World J Gastrointest Endosc. 2014; 6(6): 234–9. DOI: 10.4253/wjge.v6.i6.234 24932375 PMC4055992

[7] van Herzeele I, Aggarwal R, Neequaye S, Darzi A, Vermassen F, Cheshire NJ. Cognitive training improves clinically relevant outcomes during simulated endovascular procedures. J Vasc Surg. 2008; 48(5): 1223–30, 1230.e1. DOI: 10.1016/j.jvs.2008.06.034 18771880

[8] Khan R, Plahouras J, Johnston BC, Scaffidi MA, Grover SC, Walsh CM. Virtual reality simulation training for health professions trainees in gastrointestinal endoscopy. Cochrane Database Syst Rev. 2018; 8(8): CD008237. DOI: 10.1002/14651858.CD008237.pub3 30117156 PMC6513657

[9] Wong MAME, Chue S, Jong M, Benny HWK, Zary N. Clinical instructors’ perceptions of virtual reality in health professionals’ cardiopulmonary resuscitation education. SAGE Open Med. 2018; 6: 2050312118799602. DOI: 10.1177/2050312118799602 30245815 PMC6144504

[10] Nagendran M, Gurusamy KS, Aggarwal R, Loizidou M, Davidson BR. Virtual reality training for surgical trainees in laparoscopic surgery. Cochrane Database Syst Rev. 2013; 2013(8): CD006575. DOI: 10.1002/14651858.CD006575.pub3 23980026 PMC7388923

[11] Moro C, Štromberga Z, Raikos A, Stirling A. The effectiveness of virtual and augmented reality in health sciences and medical anatomy. Anat Sci Educ. 2017; 10(6): 549–59. DOI: 10.1002/ase.1696 28419750

[12] Bernardo A. Virtual Reality and Simulation in Neurosurgical Training. World Neurosurg. 2017; 106: 1015–29. DOI: 10.1016/j.wneu.2017.06.140 28985656

[13] Gurusamy KS, Aggarwal R, Palanivelu L, Davidson BR. Virtual reality training for surgical trainees in laparoscopic surgery. Cochrane Database Syst Rev. 2009; (1): CD006575. DOI: 10.1002/14651858.CD006575.pub2 19160288

[14] Boscolo-Berto R, Tortorella C, Porzionato A, Stecco C, Picardi EEE, Macchi V, et al. The additional role of virtual to traditional dissection in teaching anatomy: a randomised controlled trial. Surg Radiol Anat. 2021; 43(4): 469–79. DOI: 10.1007/s00276-020-02551-2 32940718 PMC8021520

[15] Ammanuel S, Brown I, Uribe J, Rehani B. Creating 3D models from Radiologic Images for Virtual Reality Medical Education Modules. J Med Syst. 2019; 43(6): 166. DOI: 10.1007/s10916-019-1308-3 31053902

[16] Cohen D, Sevdalis N, Patel V, Taylor M, Lee H, Vokes M, et al. Tactical and operational response to major incidents: feasibility and reliability of skills assessment using novel virtual environments. Resuscitation. 2013; 84(7): 992–8. DOI: 10.1016/j.resuscitation.2012.12.011 23357703

[17] Heinrichs WL, Youngblood P, Harter P, Kusumoto L, Dev P. Training healthcare personnel for mass-casualty incidents in a virtual emergency department: VED II. Prehosp Disaster Med. 2010; 25(5): 424–32. DOI: 10.1017/S1049023X00008505 21053190

[18] Dyer E, Swartzlander BJ, Gugliucci MR. Using virtual reality in medical education to teach empathy. J Med Libr Assoc. 2018; 106(4): 498–500. DOI: 10.5195/jmla.2018.518 30271295 PMC6148621

[19] Bracq M-S, Michinov E, Jannin P. Virtual Reality Simulation in Nontechnical Skills Training for Healthcare Professionals: A Systematic Review. Simul Healthc. 2019; 14(3): 188–94. DOI: 10.1097/SIH.0000000000000347 30601464

[20] Fealy S, Jones D, Hutton A, Graham K, McNeill L, Sweet L, et al. The integration of immersive virtual reality in tertiary nursing and midwifery education: A scoping review. Nurse Educ Today. 2019; 79: 14–9. DOI: 10.1016/j.nedt.2019.05.002 31078869

[21] Huang T-K, Yang C-H, Hsieh Y-H, Wang J-C, Hung C-C. Augmented reality (AR) and virtual reality (VR) applied in dentistry. Kaohsiung J Med Sci. 2018; 34(4): 243–8. DOI: 10.1016/j.kjms.2018.01.009 29655414 PMC11915632

[22] Jiang H, Vimalesvaran S, Wang JK, Lim KB, Mogali SR, Car LT. Virtual Reality in Medical Students’ Education: Scoping Review. JMIR Med Educ. 2022; 8(1): e34860. DOI: 10.2196/34860 35107421 PMC8851326

[23] Marougkas A, Troussas C, Krouska A, Sgouropoulou C. Virtual Reality in Education: A Review of Learning Theories, Approaches and Methodologies for the Last Decade. Electronics. 2023; 12(13): 2832. DOI: 10.3390/electronics12132832

[24] Kolb DA. Experimental learning: Experience as the source of learning and development. Englewood Cliffs, NJ: Prentice-Hall; 1984.

[25] Dennick R. Constructivism: reflections on twenty five years teaching the constructivist approach in medical education. Int J Med Educ. 2016; 7: 200–5. DOI: 10.5116/ijme.5763.de11 27344115 PMC4939219

[26] Krishnamurthy K, Selvaraj N, Gupta P, Cyriac B, Dhurairaj P, Abdullah A, et al. Benefits of gamification in medical education. Clin Anat. 2022; 35(6): 795–807. DOI: 10.1002/ca.23916 35637557

[27] Tyng CM, Amin HU, Saad MNM, Malik AS. The Influences of Emotion on Learning and Memory. Front Psychol. 2017; 8: 1454. DOI: 10.3389/fpsyg.2017.01454 28883804 PMC5573739

[28] Riva G, Mantovani F, Capideville CS, Preziosa A, Morganti F, Villani D, et al. Affective interactions using virtual reality: the link between presence and emotions. Cyberpsychol Behav. 2007; 10(1): 45–56. DOI: 10.1089/cpb.2006.9993 17305448

[29] Schmidt H, Becker J, Friederichs H, Geldmacher T, Marschall B, Sensmeier J, Muthny FA. Interdisciplinary Teaching Module for Transplantation – Concept, Management and Evaluation. Transplantationsmedizin. 2010; 22: 248–54.

[30] Holling M, Stummer W, Friederichs H. Teaching the concept of brain death in undergraduate medical education. J Surg Educ. 2015; 72(3): 504–8. DOI: 10.1016/j.jsurg.2014.10.012 25467732

[31] Richtlinie gemäß § 16 Abs. 1 S. 1 Nr. 1 TPG für die Regeln zur Feststellung des Todes nach § 3 Abs. 1 S. 1 Nr. 2 TPG und die Verfahrensregeln zur Feststellung des endgültigen, nicht behebbaren Ausfalls der Gesamtfunktion des Großhirns, des Kleinhirns und des Hirnstamms nach § 3 Abs. 2 Nr. 2 TPG, Fünfte Fortschreibung; [Translated titel: Directive pursuant to § 16 para. 1 sentence 1 no. 1 TPG for the rules for determining death pursuant to § 3 para. 1 sentence 1 no. 2 TPG and the procedural rules for determining the definitive, irreversible loss of total function of the cerebrum, cerebellum and brain stem pursuant to § 3 para. 2 no. 2 TPG, Fifth Update]. Deutsches Ärzteblatt Online 2022.

[32] Kratz T. Delir bei Demenz. Z Gerontol Geriatr. 2007; 40(2): 96–103. DOI: 10.1007/s00391-007-0435-5 17450409

[33] Kockwelp P, Junga A, Valkov D, Marschall B, Holling M, Risse B. Towards VR Simulation-Based Training in Brain Death Determination. In: 2022 IEEE Conference on Virtual Reality and 3D User Interfaces Abstracts and Workshops (VRW). IEEE; 2022. pp. 287–92. DOI: 10.1109/VRW55335.2022.00065

[34] Danner D, Rammstedt B, Bluemke M, Treiber L, Berres S, Soto C, et al. Die deutsche Version des Big Five Inventory 2 (BFI-2); [Translated titel: German version of BIG Five Inventory 2 (BFI-2); 2016.

[35] Mistry D, Brock CA, Lindsey T. The Present and Future of Virtual Reality in Medical Education: A Narrative Review. Cureus. 2023; 15(12): e51124. DOI: 10.7759/cureus.51124 38274907 PMC10810257

[36] Hew KF, Lo CK. Flipped classroom improves student learning in health professions education: a meta-analysis. BMC Med Educ. 2018; 18(1): 38. DOI: 10.1186/s12909-018-1144-z 29544495 PMC5855972

[37] Lee Y, Kim G, Lee KH, Park J, Kim HK. Comparison of Tutorial Methods in Virtual Reality Games for a Better User Experience. Applied Sciences. 2024; 14(16): 7141. DOI: 10.3390/app14167141

[38] Ros M, Debien B, Cyteval C, Molinari N, Gatto F, Lonjon N. Applying an immersive tutorial in virtual reality to learning a new technique. Neurochirurgie. 2020; 66(4): 212–8. DOI: 10.1016/j.neuchi.2020.05.006 32623059

[39] Duff JP, Morse KJ, Seelandt J, Gross IT, Lydston M, Sargeant J, et al. Debriefing Methods for Simulation in Healthcare: A Systematic Review. Simul Healthc. 2024; 19(1S): S112–S121. DOI: 10.1097/SIH.0000000000000765 38240623

[40] ARtillery Intelligence. Prognose zum Umsatz mit Virtual Reality weltweit in den Jahren 2021 bis 2026 (in Milliarden US-Dollar); [Translated titel: Predicted global sales growth in virtual reality from 2021 to 2026 (in billions of US dollars)]. Statista; 2022 Oct 17. Available from: URL: https://de.statista.com/statistik/daten/studie/318536/umfrage/prognose-zum-umsatz-mit-virtual-reality-weltweit/.

