# Supplementary material for: Teach the Unteachable with a Virtual Reality (VR) Brain Death Scenario – 800 Students and 3 Years of Experience
Source: Perspect Med Educ. 2025 Jan 28;14(1):44–54. doi: 10.5334/pme.1427 (PMC11784512; doi:10.5334/pme.1427)
Supplement: Supplementary Material 1. — Questionnaires used. [file pme-14-1-1427-s1.pdf]

Supplementary material 1

Pre intervention questionnaire:

1. Please enter your 6-digit study code [free text]
2. Please select your gender [list choice; “female”, “male”, “diverse”]
3. Please enter your age [free text]
4. Please enter your body size in cm [free text]
5. Please enter your preferred hand [list choice “left hand”, “right hand”]
6. Have you attended the lecture ‘Brain death diagnostics’ as part of the transplantation module? [list choice; “Yes”, “No”]
7. Do you own a VR System? [list choice; “Yes”, “No”]
8. Did you used a VR System before? [list choice; “Yes, regularly”, ”Yes, but only once or rarely”, “No”]
9. Unified Motive Scales 6 [for other study protocol]
10. Allgemeine Selbstwirksamkeits Kurzskala / General self-efficacy short-rating scale [for other study protocol]
11. Below are two statements about your subjective learning success that you more or less agree with. For each statement, please tick how much you would agree, from ‘Strongly disagree’ (1) to ‘Strongly agree’ (5) [List choice; 5-point Likert scale].
    a. I consider this teaching format to be suitable for learning brain death diagnostics
    b. I consider myself competent to perform brain death diagnostics.

Post intervention questionnaire:

1. Please enter your 6-digit study code [free text]
2. Please state honestly which reflexes you have tested or what you have seen. The information will not be passed on, graded or have any effect on your studies, but will be used for your self-reflection and to improve the application.
    a. Unconsciousness [List choice; “Yes”, “No”, “was not tested because…[free text]”]
    b. Pupils wide/medium-wide [List choice; “Left”, “Right”, “Pupils narrow”, “not testable from a medical view”, “was not tested because…[free text]”]
    c. Light reflex absent [List choice; “Left”, “Right”, “Light reflex positive”, “not testable from a medical view”, “was not tested because…[free text]”]
    d. oculocephalic/vestibulo-ocular reflex absent [List choice; “Left”, “Right”, “oculocephalic/vestibulo-ocular reflex positive”, “not testable from a medical view”, “was not tested because…[free text]”]
    e. corneal reflex absent [List choice; “Left”, “Right”, “corneal reflex positive”, “not testable from a medical view”, “was not tested because…[free text]”]
    f. pain reflex absent [List choice; “Yes, pain reflex absent”, “No, pain reflex present”, “not testable from a medical view”, “was not tested because…[free text]”]
    g. pharyngeal reflex absent [List choice; “Yes, pharyngeal reflex absent”, “No, pharyngeal reflex present”, “not testable from a medical view”, “was not tested because…[free text]”]
    h. tracheal reflex absent [List choice; “Yes, tracheal reflex absent”, “No, tracheal reflex present”, “not testable from a medical view”, “was not tested because…[free text]”]
    i. Apnoe test

        i. [List choise; “performed”, “not performed because [free text]”]
        ii. Initial pCO2 in mmHg/kPa [free text]
        iii. [List choice; “No own respiratory impulse (apnoea) detected”, “Spontaneous respiratory impulse present”]
        iv. Maximal p-CO2 in mmHg/kPa [free text]
    j. What was the final result of your investigation? Note: Please enter the result that you selected in the VR simulation. [List choice; “Brain death”, “Not brain death”]
    k. Below you can optionally leave comments on the VR test (e.g. in case of technical problems, etc.). [free text]
3. Podcast:
    a. Please rate from 1 to 6 while 6 is the best result: Did the podcast material provided in advance give you the impression that you were well prepared for using the VR hardware (headset, controller)? [6-point Likert scale]
    b. Please select the time that most accurately describes the following event: I watched the hardware podcast...[List choice; “within the last hour before the course”, “at the same day but not in the last hour before the course”, “one day before the course”, “two days before the course”, “between three days and one week before the course”, “more than one week before the course”, “not at all or incomplete”]
    c. Please rate from 1 to 6 while 6 is full agreement [List choice; 6-point Likert scale]:
            1. The hardware podcast was well organised and easy to understand.
            2. The hardware podcast didn't give me the opportunity to simply familiarise myself with the basics of hardware use.
            3. Thanks to the hardware podcast, I understood the basics of working with hardware and was quickly able to start the practical phase.
        ii. The length of the hardware podcast was: too short (1), appropriate (3), too long (5) [List choice”, “1-5”]
        iii. Please rate the hardware podcast on a scale from 1-100, while 100 is the best possible result [Slide control, 1-100]
4. Software- and Interaction Tutorial
    a. Please rate from 1 to 6 while 6 is full agreement [List choice; 6-point Likert-scale]:
        i. The tutorial was understandable and sensibly structured.
        ii. The tutorial provided an invaluable opportunity to gain familiarity with the fundamental principles of control and movement.
        iii. I felt unsafe in the training environment.
        iv. Thanks to the tutorial, I understood the basics of control and movement and was able to apply them in the course.
    b. The length of the Software- and Interaction Tutorial was: too short (1), appropriate (3), too long (5) [List choice”, “1-5”]
    c. Please rate the Software- and Interaction Tutorial on a scale from 1-100, while 100 is the best possible result [Slide control, 1-100]
5. Big Five Personality Test [for other study protocol]
6. I felt uncomfortable in virtual reality and experienced unpleasant symptoms such as nausea, dizziness and headaches. [List choice; 5-point Likert-scale]
7. Please rate the entire VR application on a scale of 1-100, where 100 is the best possible result. [Slide control, 1-100]

Post intervention and following course questionnaire:

1. Please enter your 6-digit study code [free text]
2. Below are several statements about your subjective learning success that you more or less agree with. For each statement, please tick how much you would agree, from ‘Strongly disagree’ (1) to ‘Strongly agree’ (5). Please refer to the entire learning unit of this day when making your statements. [List choice, 6-point Likert-scale]
    a. I consider this teaching format to be suitable for learning brain death diagnostics
    b. I consider myself competent to perform brain death diagnostics.
    c. I consider the skills I have learnt for carrying out brain death diagnostics to be useful.
    d. Investing time in this learning unit was worthwhile.
    e. I think I will still be able to report on what I have learnt some time after the learning unit.
    f. In the learning unit, I had the opportunity to try out things that I would later have to implement in my work.
    g. The learning unit was challenging in a stimulating way.
    h. The learning unit realistically depicted the challenges of brain death diagnostics
3. Here you have the opportunity to give your personal feedback to improve the course. Thank you very much! [free text]
