# Supplementary material for: Teach the Unteachable with a Virtual Reality (VR) Brain Death Scenario – 800 Students and 3 Years of Experience
Source: Perspect Med Educ. 2025 Jan 28;14(1):44–54. doi: 10.5334/pme.1427 (PMC11784512; doi:10.5334/pme.1427)
Supplement: Supplementary Material 2. — Task sheet VR course. [file pme-14-1-1427-s2.pdf]

Citrus

As a specialist in neurosurgery, you are called to see a patient.

Carry out brain death diagnostics.

You will find all necessary equipment in the patient's room.

Patient: Lena Malec

Born: 13 May 1994

Post quad accident 3 days ago, since then continuously intubated, ventilated

Severe craniocerebral trauma, initial trephination drilling

Serial rib fracture of left ribs IIX-X

Grade II splenic rupture according to AAST - conservative management

Fracture of the left lateral zygomatic arch

Fracture of the right tibial plateau, pCSM intact - primary splinting

Hematoma at the level of the right Spina illiaca

Multiple contusions and abrasions on the trunk and extremities

All anesthetic medication was discontinued as per the requirements.
