# Supplementary material for: Teach the Unteachable with a Virtual Reality (VR) Brain Death Scenario – 800 Students and 3 Years of Experience
Source: Perspect Med Educ. 2025 Jan 28;14(1):44–54. doi: 10.5334/pme.1427 (PMC11784512; doi:10.5334/pme.1427)
Supplement: Supplementary Material 3. — Table of adjustments during time. [file pme-14-1-1427-s3.pdf]

	Rooms	Monitoring	Hardware	Course material	Software
Pre-VR	12 identical rooms with separate workspaces	Video monitoring for real rooms + Venetian mirror<br><br>Intercom system for real rooms	Basic computer and regarding standard hardware	non	non
Pilot	6 rooms updated<br><br>Addition of touch latch to the PC cabinets to improve the cooling air supply<br><br>Installation of additional sockets in the ceiling area for the tracking hardware	Additional screens to mirror the students field-of-view in VR	6 high-performance computers (includes Ryzen 5 5600X, RTX 3070 and 32 GB RAM)<br><br>6 head mounted displays (HMDs) of the model "Valve Index" by Steam	Paper based task<br><br>Software-based tutorial adapted to the required interactions<br><br>Pre-course podcast for additional content-based preparation	Intensive care unit with young female patient, intubated. Working monitoring system and blood gas analysis system so as examination utensiles
Curricular course	Structural changes to the computer cabinets to further improve air circulation<br><br>Plug-in module installed to reduce wear and tear on the HMD connections	central monitoring system for remote vision and control of all software applications<br><br>Function for requesting (technical-)help within the software	12 high-performance computers (includes Ryzen 5 5600X, RTX 3070 and 32 GB RAM)<br><br>12 head mounted displays (HMDs) of the model "Valve Index" by Steam + one backup headset	Digital presented task<br><br>Software-based tutorial adapted to the required interactions<br><br>Pre-course podcast for additional content- **and** hardware-based preparation	See above plus visible hematomas including bandages<br><br>several leads according to ICU patients (IV access, syringe pump)<br><br>multiple personal belongings (get well soon card, teddy bear)
